# Oral Health, Oral Microbiota, and Incidence of Stroke-Associated Pneumonia—A Prospective Observational Study

**DOI:** 10.3389/fneur.2020.528056

**Published:** 2020-11-06

**Authors:** Fabian Cieplik, Alma Maria Wiedenhofer, Verena Pietsch, Karl-Anton Hiller, Andreas Hiergeist, Andrea Wagner, Dobri Baldaranov, Ralf A. Linker, Jonathan Jantsch, Wolfgang Buchalla, Felix Schlachetzki, André Gessner

**Affiliations:** ^1^Department of Conservative Dentistry and Periodontology, University Hospital Regensburg, Regensburg, Germany; ^2^Institute of Clinical Microbiology and Hygiene, University Hospital Regensburg, Regensburg, Germany; ^3^Department of Neurology, University of Regensburg, Regensburg, Germany

**Keywords:** oral health, oral microbiota, stroke, stroke care, pneumonia, stroke-associated pneumonia

## Abstract

Stroke-associated pneumonia is a major cause for poor outcomes in the post-acute phase after stroke. Several studies have suggested potential links between neglected oral health and pneumonia. Therefore, the aim of this prospective observational study was to investigate oral health and microbiota and incidence of pneumonia in patients consecutively admitted to a stroke unit with stroke-like symptoms. This study involved three investigation timepoints. The baseline investigation (within 24 h of admission) involved collection of demographic, neurological, and immunological data; dental examinations; and microbiological sampling (saliva and subgingival plaque). Further investigation timepoints at 48 or 120 h after baseline included collection of immunological data and microbiological sampling. Microbiological samples were analyzed by culture technique and by 16S rRNA amplicon sequencing. From the 99 patients included in this study, 57 were diagnosed with stroke and 42 were so-called stroke mimics. From 57 stroke patients, 8 (14%) developed pneumonia. Stroke-associated pneumonia was significantly associated with higher age, dysphagia, greater stroke severity, embolectomy, nasogastric tubes, and higher baseline C-reactive protein (CRP). There were trends toward higher incidence of pneumonia in patients with more missing teeth and worse oral hygiene. Microbiological analyses showed no relevant differences regarding microbial composition between the groups. However, there was a significant ecological shift over time in the pneumonia patients, probably due to antibiotic treatment. This prospective observational study investigating associations between neglected oral health and incidence of SAP encourages investigations in larger patient cohorts and implementation of oral hygiene programs in stroke units that may help reducing the incidence of stroke-associated pneumonia.

## Introduction

Stroke was found to be the second leading cause of death worldwide after ischemic heart disease in 2016, accounting for 5.5 million attributable deaths (1), and the estimated global lifetime risk of stroke was 24.9% for those aged 25 or older in 2016 (2). Thus, stroke-related treatment costs (particularly post-stroke care) represent a major economic burden for public health care (3).

Infections are common complications in the acute phase after stroke (4–6). In particular, stroke-induced immunodepression, a systemic anti-inflammatory response syndrome, relates to higher susceptibility to infections (6, 7). In a systematic review and meta-analysis from 87 studies involving more than 137,000 patients, Westendorp et al. found that infections complicated acute stroke in 30% of the cases with pneumonias and urinary tract infections (UTIs) being most common (6). In particular, stroke-associated pneumonia has been found as a major cause for poor outcomes resulting in death following stroke, increasing the 30-day mortality rate threefold and also expanding related costs, length of stay, and likelihood of poor outcomes (6–8).

Micro-aspiration is known to be the major pathogenetic mechanism of most pneumonias (9). Therefore, the risk of developing stroke-associated pneumonia is increased in patients suffering from dysphagia, which is a common condition following stroke with incidence rates between 37 and 78% reported in the literature (10, 11). While 13–48% of dysphagic stroke patients develop pneumonia, up to half of stroke patients have no history of swallowing problems before developing pneumonia (11). Therefore, other contributing risk factors for stroke-associated pneumonia should be considered besides dysphagia (11, 12). Furthermore, there are other stroke-related functional impairments (such as weakened buccal musculature, oral stereognosis, hypotonic lip musculature, or oral apraxia) that may lead to oral hygiene issues (13). Accordingly, Pow et al. showed that stroke survivors had significantly higher plaque and bleeding scores at hospital discharge as compared with community-dwelling elderly people (14).

Ample evidence exists supporting a potential link between oral diseases or insufficient oral hygiene and development of aspiration pneumonia, although not specifically considering stroke patients (12, 15–17) and not from prospective investigations (18). In contrast, some studies have shown that improvement of oral care regimens in stroke patients can reduce incidence of stroke-associated pneumonia (18–21). Boaden *et al*. recently described the bacterial profiles of the oral cavities of 50 patients during the first 2 weeks following stroke but found no associations between particular phylotypes and infections, concluding that their study may support larger observational studies (19).

Therefore, the aim of this prospective observational study was to investigate dental and oral health, oral microbiota, neurological and immunological parameters, and incidence of stroke-associated pneumonia in patients consecutively admitted to a stroke unit presenting stroke-like symptoms.

## Methods

### Study Design

The present study is a prospective observational study investigating dental and oral health, oral microbiota, neurological parameters, and incidence of pneumonia in patients consecutively admitted to a stroke unit (Department of Neurology, Bezirksklinikum Regensburg & University of Regensburg, Regensburg, Germany) with stroke-like symptoms during a study period of 5 months starting in February 2018.

Patients were included in this study if they showed symptoms of acute stroke and were recruited within 24 h of admission. Exclusion criteria were an indication for endocarditis prophylaxis, intubation at time of inpatient admission, and cases of stroke recurrence within 1 month. Patients were screened for eligibility by two dentists (AMW and VP) and were provided with a detailed description of the study outline which involved three investigation timepoints: The baseline (BL) investigation had to be within 24 h of inpatient admission and involved documentation of demographic, neurological, and immunological data; dental examinations; and microbiological sampling; the second and third investigation timepoints at 48 h after BL or 120 h after BL, respectively, included collecting immunological data and microbiological sampling. Patients discharged from inpatient care earlier than the 48- or 120-h investigation contributed data from the preceding timepoints only. Written informed consent was obtained from all individual participants included in the study. All data were collected by two dentists (AMW and VP) with support from two experienced neurologists (AW and DB).

The study design was approved by the internal review board of the University of Regensburg (ref. 17-806-101) in accordance with the 1964 Helsinki declaration and its later amendments or comparable ethical standards. The study has been registered at the German Clinical Trials Register (ref. DRKS00018063).

### Documentation of Demographic, Neurological, and Immunological Data

Demographic and neurological data, age, sex, smoking habits, medical history, comorbidities, and medications of the patients were recorded. Stroke was diagnosed according to the definition given by Sacco et al. (20), that is, either with direct evidence from neuroimaging or clinical stroke lasting >24 h. In addition to the Trial of Org 10,172 in Acute Stroke Treatment (TOAST) classification (21), modified Rankin scale (mRS) (22), and National Institutes of Health Stroke Scale (NIHSS) (23, 24), stroke was differentiated into ischemic and hemorrhagic and according to localization of the stroke. Dysphagia diagnoses were carried out in a two-step procedure. First, the so-called Standardized Swallowing Assessment (SSA) as described by Perry (25, 26) was performed as an initial dysphagia screening tool by specialized nurses of the stroke unit team. Then, specialized speech therapists performed detailed clinical swallowing examinations (CSE; Klinische Schluckuntersuchung, KSU) (27), on the basis of which dysphagia diagnoses were made. CSE is based on inspection of mouth and pharynx, reflex tests (coughing, gag reflex, and swallowing reflex), inspection of voluntary movements, sensibility tests, and swallowing tests (for porridge, water, and solid food) like the swallow provocation test (SPT) or the cough reflex test (CRT) (27). Pneumonia was diagnosed according to Mann criteria (28), and UTI was identified through positive microbiological cultures or negative cultures with leukocytosis. Immunological data were recorded at BL and at 48 and 120 h after BL for assessing the inflammatory markers C-reactive protein (CRP) and leukocyte count.

### Dental Examinations

All dental examinations were performed bedside by two dentists (AMW and VP) that had been calibrated to an experienced general dentist (FC) before (κ > 0.79). Dental caries was recorded at the cavitation stage only (29). Teeth were charted as missing, healthy, decayed, root remnants, or restored, and the decayed, missing, and filled teeth (DMFT) index was calculated (30). Oral hygiene was assessed by means of the full-mouth approximal plaque index (API) (31). Probing pocket depths (PPDs) were measured at six points for each tooth as the distance between the gingival margin and fundus of a periodontal pocket, and the deepest pocket (PPD_max_) was recorded for each tooth. The regularity of dental examinations and recall visits at the dental practice was asked from each patient.

### Microbiological Sampling

Microbiological sampling was performed at BL and (if applicable) at 48 and 120 h after BL by sampling the tongue dorsa and subgingival plaque. In cases of edentulism, samples were taken from alveolar ridges instead of subgingival plaque. All samples were taken as duplicates, one for culture-dependent analysis by using the Port-A-Cul™ transport system (Becton Dickinson, Franklin Lakes, NJ, United States) for subgingival plaque or the eSwab system (Hain Lifescience, Nehren, Germany) for tongue and alveolar ridge samples, and the other was stored at −80°C by using the eNat™ system (Copan Diagnostics, Murrieta, CA, United States) for later 16S rRNA amplicon sequencing.

Subgingival plaque was collected from the two deepest sites in each patient (as identified in the dental examination) by introducing sterile paper points (Roeko paper points ISO 35, Coltene, Altstätten, Switzerland) into the periodontal pockets for 10 s and was stored in Port-A-Cul™ or eNat™, respectively.

Microbiological samples from the tongue dorsa and subgingival plaque or alveolar ridges, respectively, were then pooled for each patient for all further culture-dependent and culture-independent analyses.

### Culture-Dependent Analysis by Means of MALDI-TOF MS

Microbial samples for culture-dependent analysis were suspended in thioglycolate broth, which was then plated on boiled blood, Columbia blood, McConkey blood, Schaedler, Schaedler kanamycin–vancomycin (KV), and Wilkins-Chalgren (WC) agar plates. Boiled blood, Columbia blood, and McConkey blood agar plates were incubated aerobically at 35°C for 48 h, while Schaedler, Schaedler KV, and WC agar plates were incubated anaerobically at 35°C for 120 h. After these respective culture periods, plates were analyzed for bacterial growth. Bacterial isolates were discriminated according to their colony morphologies and identified to the species level by means of matrix-assisted laser desorption ionization-time of flight mass spectrometry (MALDI-TOF MS) employing a Microflex mass spectrometer and BioTyper analysis software (both from Bruker, Billerica, MA, United States). Results were summarized on a genus level and were separately shown for each group and each timepoint as relative proportions of patients carrying a certain genus.

### 16S rRNA Amplicon Sequencing

Samples in eNat preservation buffer were thawed, and a total of 250 μl of stabilized material was subjected to repeated bead beating on the TissueLyser II instrument (Qiagen, Hilden, Germany) using 0.1-mm silica spheres (MP Biomedicals, Irvine, CA United States) supplemented with two 1.4-mm stainless steel beads (Sigma-Aldrich, St. Louis, MI, United States). Nucleic acids were purified from prepared lysates by means of the MagNA Pure 96 instrument using the MagNA Pure 96 DNA and Viral NA Large Volume Kit (Roche Molecular Diagnostics, Mannheim, Germany).

The bacterial load in extracted nucleic acids was determined by qPCR quantification of 16S rRNA gene copy numbers on the LightCycler 480 II instrument. PCRs included universal eubacterial 16S rRNA gene primers 341F and 785R together with FAM-labeled universal hydrolysis probe 506TM and the LightCycler 480 Probes Master kit (Roche Molecular Diagnostics, Mannheim, Germany). Nucleic acid extracts from matching tongue dorsa and subgingival plaque samples for each patient were pooled using 5e+6 16S rRNA gene copies each.

V1 to V3 hypervariable regions of bacterial 16S rRNA genes were amplified from a total of 1e+7 bacterial 16S rDNA copies using the forward primer S-D-Bact-0008-c-S-20 containing a 10-bp barcode sequence and the reverse primer S-D-Bact-0517-a-A-18. The resulting amplicons were purified with MagSi-NGS^PREP^ Plus beads (Steinbrenner Laborsysteme, Wiesenbach, Germany). Copy numbers of amplicons containing A and P1 adaptors were determined using the KAPA Library Quantification Ion Torrent Kit (Roche Diagnostics, Mannheim, Germany). To prepare the DNA library, equimolar concentrations of adaptor-labeled amplicons were pooled to reach a final library pool concentration of 100 pM. This library was re-amplified by isothermal amplification using the Ion PGM Template IA 500 Kit (Thermo Fisher Scientific, Darmstadt, Germany) and thereafter subjected to sequencing on an Ion Torrent™ PGM (Thermo Fisher Scientific, Darmstadt, Germany).

Signal processing and base calling was performed using the Torrent Suite Software version 5.10 with switched-off quality trimming. Resulting fastq files were transferred from the Torrent Server. Adapter and 16S primer sequences as well as reads with an average quality below 15 were removed from processed reads using cutadapt 1.16. Low-quality bases were trimmed from 3′ ends in a sliding-window approach using sickle version 1.33 (32) with a quality cutoff of 15 and length cutoff of 251 bases. Cutadapt (33) was used for demultiplexing of filtered reads, allowing no errors. Demultiplexed reads were further processed with vsearch version 2.9.1 (34). Only reads with more than five expected errors were subjected to denoising of reads using the unoise3 algorithm with a minimum cluster size of 3 and alpha value of 2. Chimeras were filtered *de novo* using uchime3. Remaining reads were clustered to centroids at 98.5% sequence identity and mapped back to unfiltered reads by means of the cluster_fast and usearch_global algorithms, respectively. SINTAX was used to annotate taxonomies of cluster centroids by searching against a modified SILVA release 132 reference database.

A BIOM-formatted operational taxonomic unit (OTU) table was imported into R, and UniFrac distances (35) were calculated using the phyloseq package (36). Pairwise multilevel comparisons were made using the Adonis function of vegan 2.5. Significantly altered taxa between groups were assessed with the linear discriminant analysis effect size (LefSE) (37). Differentially abundant species between BL and 120 h after BL were analyzed with DESeq2 version 1.24 (38).

### Data Analysis

All results are shown as medians including first and third quartiles or relative proportions (numbers of patients), respectively. Data were analyzed statistically by applying nonparametric procedures (Mann–Whitney *U* or χ^2^-tests, respectively), for pairwise comparisons between groups on an α = 0.05 level of significance. For evaluation of a general influence of a given parameter on all groups, the level of significance was adjusted to α^*^(*k*) = 1–(1–α)^1/k^ (*k* = number of pairwise tests) according to the error rates method (39), yielding an α^*^(3) = 0.01695243 for the combination of three pairwise tests. All calculations were performed using SPSS v. 25 (SPSS Inc., Chicago, IL, United States).

## Results

### Patient Inclusion and Allocation to Groups

From 415 patients admitted to the stroke unit, 99 (23.9%) met the inclusion criteria and agreed with participation in this study. From these, 57 were diagnosed with ischemic stroke while 42 presented without diagnosis of stroke (so-called “stroke mimics”). Eight out of 57 stroke patients (14%) developed pneumonia. Accordingly, the 99 included patients were allocated to three groups:

- Stroke mimic group: patients with stroke-like symptoms who were not diagnosed with stroke.
- Stroke group: patients diagnosed with stroke who did not develop pneumonia.
- Stroke-associated pneumonia group: patients diagnosed with stroke who developed pneumonia.

**Figure 1:**
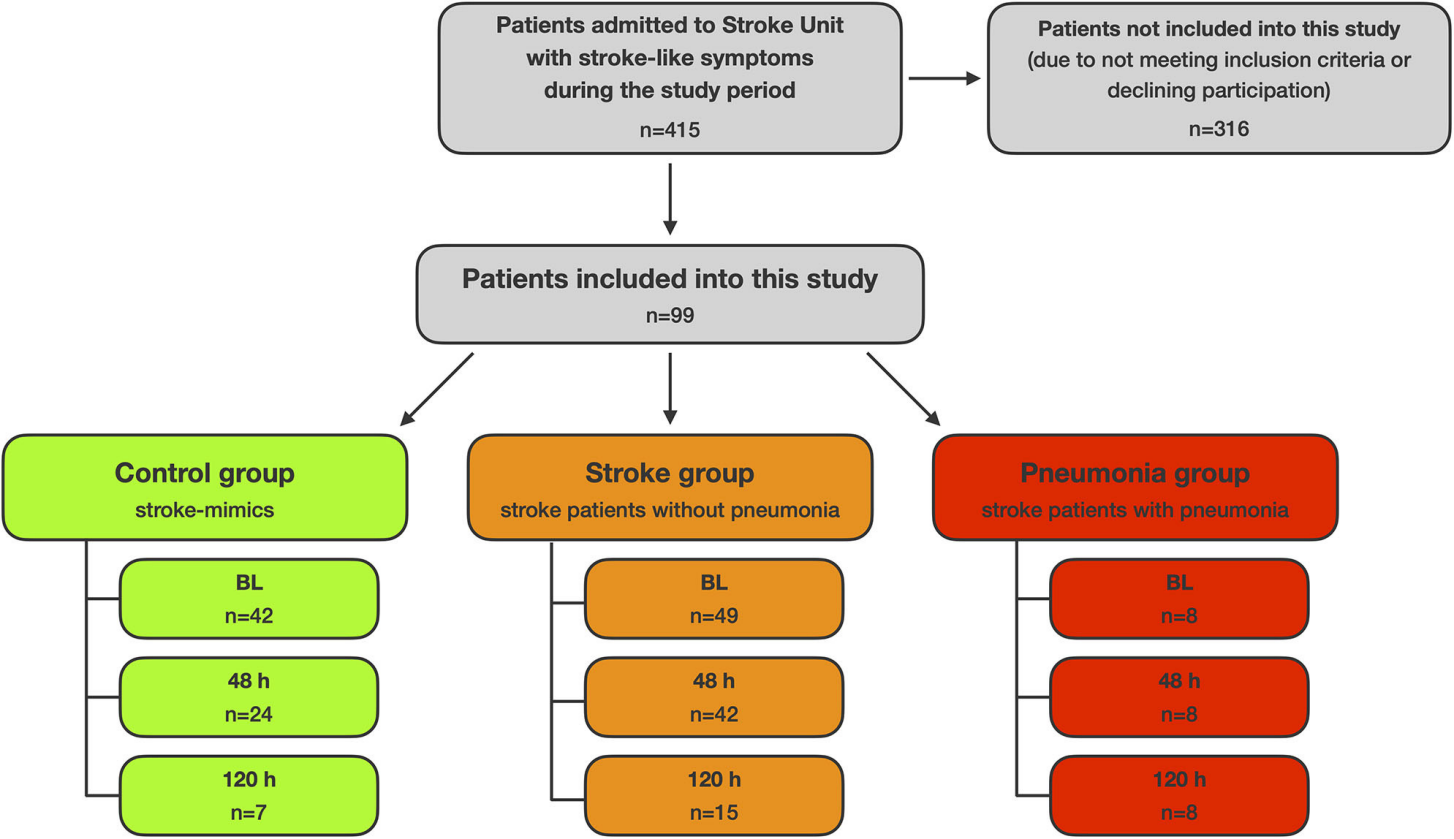
Flow of patients.

Figure 1 shows the flow of patients throughout the stages of this study.

### Demographic and Neurological Data

Patients developing stroke-associated pneumonia were significantly older (median age 79.5 years) compared to stroke mimics (66.5) and stroke patients without pneumonia (40) (Table 1). Pneumonia patients also had significantly longer inpatient stays (median 10 days) and suffered significantly more often from dysphagia (75%) compared with stroke mimics (3 days; 9.5%) and stroke patients (4 days; 12.2%). Stroke severity was significantly higher in pneumonia patients than in stroke patients without pneumonia (median NIHSS 6.5 vs. 2). Likewise, mRS was significantly higher in pneumonia patients (median mRS 3.5 at admission and 3 at discharge) as compared to stroke patients without pneumonia (mRS 2 and 1) and stroke mimics (mRS 1, both) with the latter being significantly different, too. Embolectomy and application of nasogastric tubes were significantly more often in pneumonia patients (62.5% both) than in stroke patients without pneumonia (4.1% both). General influences among all groups were found according to the error rates method for the parameters sex, inpatient stay, dysphagia, mRS, history of stroke, and nasogastric tube.

**Table 1:** Demographic, neurological, and infection data.

**Parameter**		**C (*n* = 42)**	**S (*n* = 49)**	**P (*n* = 8)**	**Significant differences**		
					**C vs. S**	**C vs. P**	**S vs. P**
Age^a^	[years]	66.5 (51.3; 78.3)	71 (59; 78)	79.5 (71.8; 85.8)	–	0.022	0.038
Sex^b^	male	42.9% (18)	69.4% (34)	62.5% (5)	0.010	–	–
Inpatient stay^a^	[days]	3 (2; 4)	4 (3; 7)	10 (9; 15.3)	0.004	0.000	0.000
Dysphagia^b^		9.5% (4)	12.2% (6)	75% (6)	–	0.000	0.001
NIHSS^a^	at inpatient admission	–^§^	2 (1; 4)	6.5 (3.3; 18.5)	n.d.	n.d.	0.008
mRS^a^	at inpatient admission	1 (1; 2)	2 (2; 3)	3.5 (3; 5)	0.002	0.000	0.001
	at inpatient dismissal	0.5 (0; 1)	1 (1; 2)	3 (2.3; 3.8)	0.002	0.000	0.000
Stroke localization^b^	anterior circulation	–^§^	61.2% (30)	62.5% (5)	n.d.	n.d.	–
	posterior circulation	–^§^	38.8% (19)	37.5% (3)			
Stroke localization^b^	right	–^§^	46.9% (23)	62.5% (5)	n.d.	n.d.	–
	left	–^§^	46.9% (23)	25% (2)			
	both sides	–^§^	6.1% (3)	12.5% (1)			
TOAST classification^b^	microangiopathic	–^§^	18.4% (9)	0% (0)	n.d.	n.d.	0.030
	macroangiopathic	–^§^	42.9% (21)	12.5% (1)			
	cardioembolic	–^§^	28.6% (14)	75% (6)			
	undetermined	–^§^	8.2% (4)	0% (0)			
	other	–^§^	2% (1)	12.5% (1)			
Comorbidities^a^	[*n*]	2 (1; 4.3)	3 (2; 5)	3.5 (2.3; 5.5)	–	–	–
Medications^a^	[*n*]	2.5 (1; 4)	2 (1; 4)	2 (1.3; 2.8)	–	–	–
Active smoking status^b^		38.1% (16)	49% (24)	50% (4)	–	–	–
History of stroke^b^		45.2% (19)	16.3% (8)	25% (2)	0.003	–	–
History of pneumonia^b^	yes	26.2% (11)	6.1% (3)	0% (0)	–	–	–
	unknown	2.4% (1)	4.1% (2)	0% (0)			
Embolectomy^b^		–^§^	4.1% (2)	37.5% (3)	n.d.	n.d.	0.017
Nasogastric tube^b^		0% (0)	4.1% (2)	37.5% (3)	–	0.003	0.017
Time to diagnosis of pneumonia^a^	[days]	–^$^	–^$^	2 (0.3; 3.8)	n.d.	n.d.	n.d.
UTI during inpatient care^b^^,^^**^		14.3% (6)	14.9% (7)	37.5% (3)	–	–	–
Time to diagnosis of UTI^a^^,^^***^	[days]	1 (1; 1.3)	1 (1; 5)	1 (0; 2)	–	–	–

*C, stroke mimics; S, stroke patients without pneumonia; P, stroke patients with pneumonia; NIHSS, National Institutes of Health Stroke Score; mRS, modified Rankin scale; TOAST, Trial of Org 10,172 in Acute Stroke Treatment; UTI, urinary tract infection*.

Depiction of medians^a^ (first quartile; third quartile) or relative proportions^b^ (numbers of patients) and statistically significant differences from pairwise comparisons (Mann–Whitney U or χ^2^-tests, respectively; α = 0.05). For evaluation of the general influence of a given parameter among all groups, α was adjusted according to the error rates method to α*(3) = 0.01695243.

§ *not applicable for group C*;

$ *not applicable for groups C and S; n.d., not determined*;

** *only applicable for 47 patients from group S (two patients already with UTI at time of inpatient admission)*;

*** *only applicable for six, seven, and three patients from groups C, S, and P, respectively, who developed a UTI; p-value, significant (p ≤ 0.05); –, not significant (p > 0.05)*.

### Immunological Data

Stroke-associated pneumonia patients showed a CRP increase from admission (median 3.2 mg/L) to BL (10.7 mg/L) and 48 h (52.3 mg/L), decreasing again at 120 h (37.2 mg/L) (Table 2). CRP was found significantly higher in the pneumonia group as compared to the stroke mimic group at BL and at 48 and 120 h and to the stroke without pneumonia group at 48 and 120 h. There was a general influence of the parameter CRP at BL and at 48 and 120 h among all groups according to the error rates method. Leukocyte counts were also slightly higher in the pneumonia group, which was found significantly different compared to the two other groups at 48 h.

**Table 2:** Immunological data.

**Parameter**	**Timepoint**	**C (*n* = 42)**	**S (*n* = 49)**	**P (*n* = 8)**	**Significant differences**		
					**C vs. S**	**C vs. P**	**S vs. P**
CRP (mg/L)	inpatient admission	3.2 (1.9; 4.6)	3.2 (1.7; 6.8)	3.2 (1; 8.8)	–	–	–
	BL	2.5 (0.8; 4.2)	3.2 (1.1; 8.2)	10.7 (2.4; 26.5)	–	0.016	–
	48 h	1.3 (0.7; 8.5)	3.5 (1; 10.9)	52.3 (45; 96.4)	–	0.000	0.000
	120 h	2.2 (1; 7.2)	13.1 (2.3; 20)	37.2 (23.4; 80.3)	0.017	0.000	0.001
Leukocyte counts (×1,000/μl)	inpatient admission	7.8 (6.2; 9.1)	7.5 (6.3; 9.7)	9.4 (7.8; 11.9)	–	–	–
	BL	6.9 (5.7; 9.2)	7.9 (6.5; 9.6)	8.1 (6.1; 11.4)	–	–	–
	48 h	7.7 (6.3; 8.7)	7.5 (6.2; 8.4)	10.1 (7.8; 15.8)	–	0.041	0.043
	120 h	8.1 (5.7; 11.9)	7 (5.8; 8.9)	9.8 (6.5; 13.9)	–	–	–

*C, stroke mimics; S, stroke patients without pneumonia; P, stroke patients with pneumonia; CRP, C-reactive protein*.

Depiction of medians (first quartile; third quartile) and statistically significant differences from pairwise comparisons (Mann-Whitney U tests; α = 0.05). For evaluation of the general influence of a given parameter among all groups, α was adjusted according to the error rates method to α*(3) = 0.01695243.

*p-value, significant (p ≤ 0.05); –, not significant (p > 0.05)*.

### Dental Data

Median DMFT (decayed, missing, and filled teeth) index (30) ranged between 23 and 25 without significant differences among the groups (Table 3). Despite no significant differences for numbers of decayed teeth, root remnants, and restored teeth, pneumonia patients had significantly more missing teeth (median 22) than patients from group C (median 9) and also more missing teeth than group S (median 10, not significant). About two thirds of all patients stated to have regular recall for dental examinations. API was found to be significantly higher in stroke (median 62.5%) and stroke-associated pneumonia patients (100%) as compared to stroke mimics (40%). According to the error rates method, there was a general influence of the parameter API among all groups. No significant differences in PPD_max_ were found between the groups (median between 3 and 5 mm).

**Table 3:** Dental and oral health.

**Parameter**		**C (n = 42)**	**S (*n* = 49)**	**P (*n* = 8)**	**Significant differences**		
					**C vs. S**	**C vs. P**	**S vs. P**
Decayed/missing/filled teeth (DMFT)^a^	[*n*]	23.5 (18; 26.3)	23 (17.5; 28)	25 (23.5; 27.8)	–	–	–
Decayed teeth^a^	[*n*]	0 (0; 0.3)	0 (0; 1)	0 (0; 4)	–	–	–
Root remants^a^	[*n*]	0 (0; 0)	0 (0; 0)	0 (0; 1.5)	–	–	–
Missing teeth^a^	[*n*]	9 (5.5; 16.5)	10 (6; 25)	22 (11.3; 25.8)	–	0.041	–
Restored teeth^a^	[*n*]	14 (9.5; 20)	11 (5; 17)	5 (0.3; 16.3)	–	–	–
Edentulous (full denture wearers)^b^	yes	7.1% (3)	12.2% (6)	0% (0)	–	–	–
Regular recall^b^	yes	78.6% (33)	73.5% (36)	62.5% (5)	–	–	–
API^a^^,^^*^	[%]	40 (19.4; 67.7)	62.5 (35.5; 100)	100 (66.1; 100)	0.014	0.004	–
${\text{PPD}}_{\text{max}}^{\text{a},\text{\#}}$	[mm]	4 (2; 6)	5 (2; 6)	3 (3; 7)	–	–	–

*C, stroke mimics; S, stroke patients without pneumonia; P, stroke patients with pneumonia; API, approximal plaque index; PPD_max_, maximum probing pocket depth*.

Depiction of medians^a^ (first quartile; third quartile) or relative proportions^b^ (numbers of patients) and statistically significant differences from pairwise comparisons (Mann–Whitney U or χ^2^-tests, respectively; α = 0.05). For evaluation of the general influence of a given parameter among all groups, α was adjusted according to the error rates method to α*(3) = 0.01695243.

* *measured in 39, 43, and 8 patients from groups C, S, and P, respectively*;

^#^*measured in 39, 43, and 7 patients from groups C, S, and P, respectively; p-value, significant (p ≤ 0.05); –, not significant (p > 0.05)*.

### Microbiological Data: Culture-Dependent Analysis

**Figure 2:**
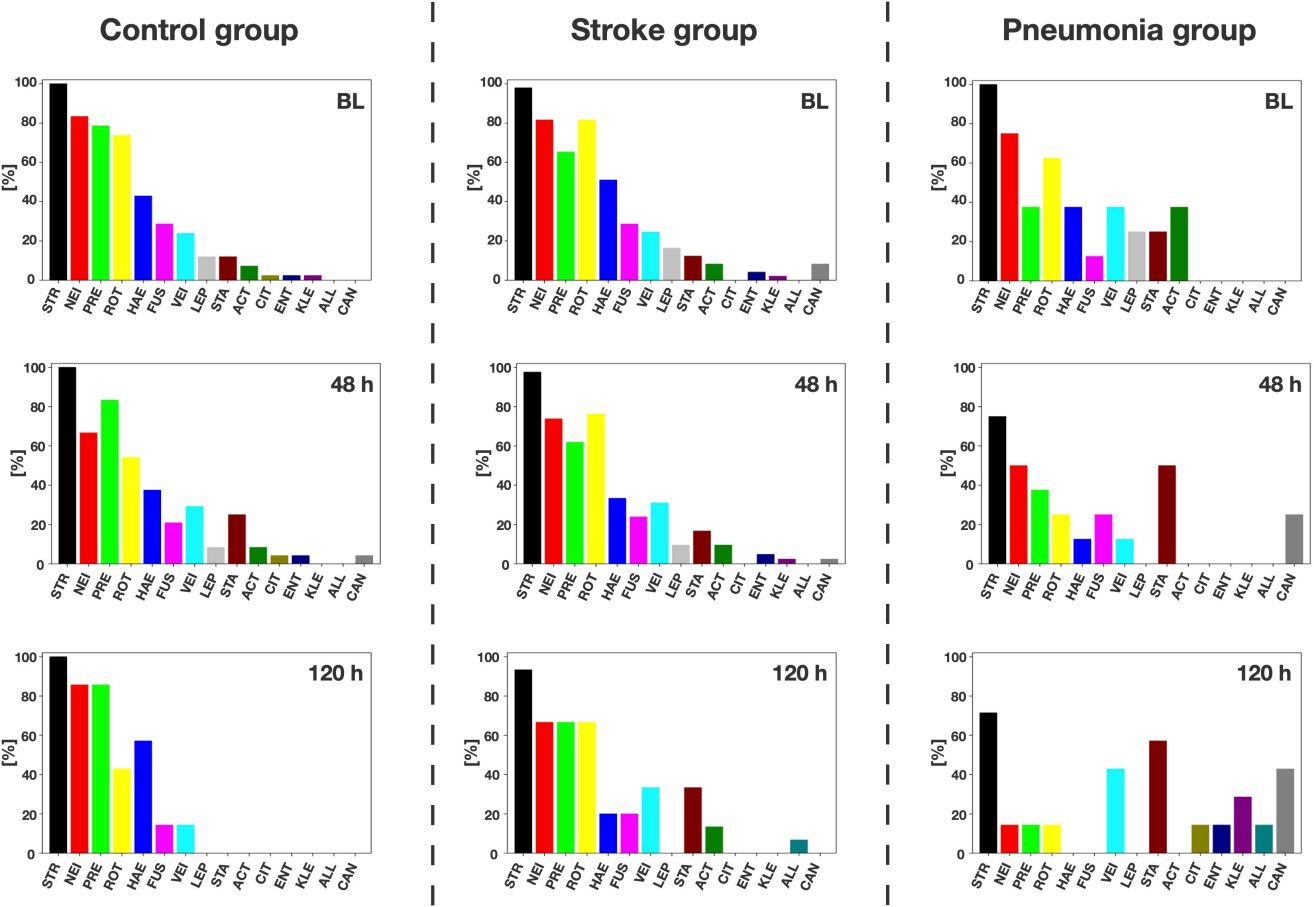
Culture-dependent analysis. Genera detected by culture-dependent matrix-assisted laser desorption ionization-time of flight mass spectrometry (MALDI-TOF MS) analysis. Relative abundancies of the 10 most abundant genera overall and the five most abundant genera additionally found in pneumonia patients at 120 h after baseline are depicted, per patient group and investigation timepoint (STR, *Streptococcus*; NEI, *Neisseria*; PRE, *Prevotella*; ROT, *Rothia*; HAE, *Haemophilus*; FUS, *Fusobacterium*; VEI, *Veillonella*; LEP, *Leptotrichia*; STA, *Staphylococcus*; ACT, *Actinomyces*; CIT, *Citrobacter*; ENT, *Enterococcus*; KLE, *Klebsiella*; ALL, *Alloscardovia*; CAN, *Candida*).

Culture-dependent analysis by MALDI-TOF MS yielded 108 distinct taxa with *Rothia mucilaginosa* (detected in 76.8% of patients), viridans streptococci (75.8%), *Prevotella melaninogenica* (74.7%), *Streptococcus oralis* (61.6%), and *Neisseria* spp. (59.6%) found most often. Summarizing taxa on the genus level yielded 33 genera (Figure 2). At BL, no clear differences could be observed between the groups regarding relative abundancies of the genera. In stroke mimics and stroke patients without pneumonia, relative abundancies stayed relatively stable over time with only *Rothia* spp. decreasing >20%. In contrast, the stroke-associated pneumonia group was subject to some ecological changes over time with decreases in relative abundancies of *Streptococcus, Neisseria, Prevotella, Rothia, Haemophilus*, and *Leptotrichia* spp. by >20% each. On the other hand, relative abundancies of *Staphylococcus, Klebsiella*, and *Candida* spp. increased by >20% each.

### Microbiological Data: 16S rRNA Amplicon Sequencing

**Figure 3:**
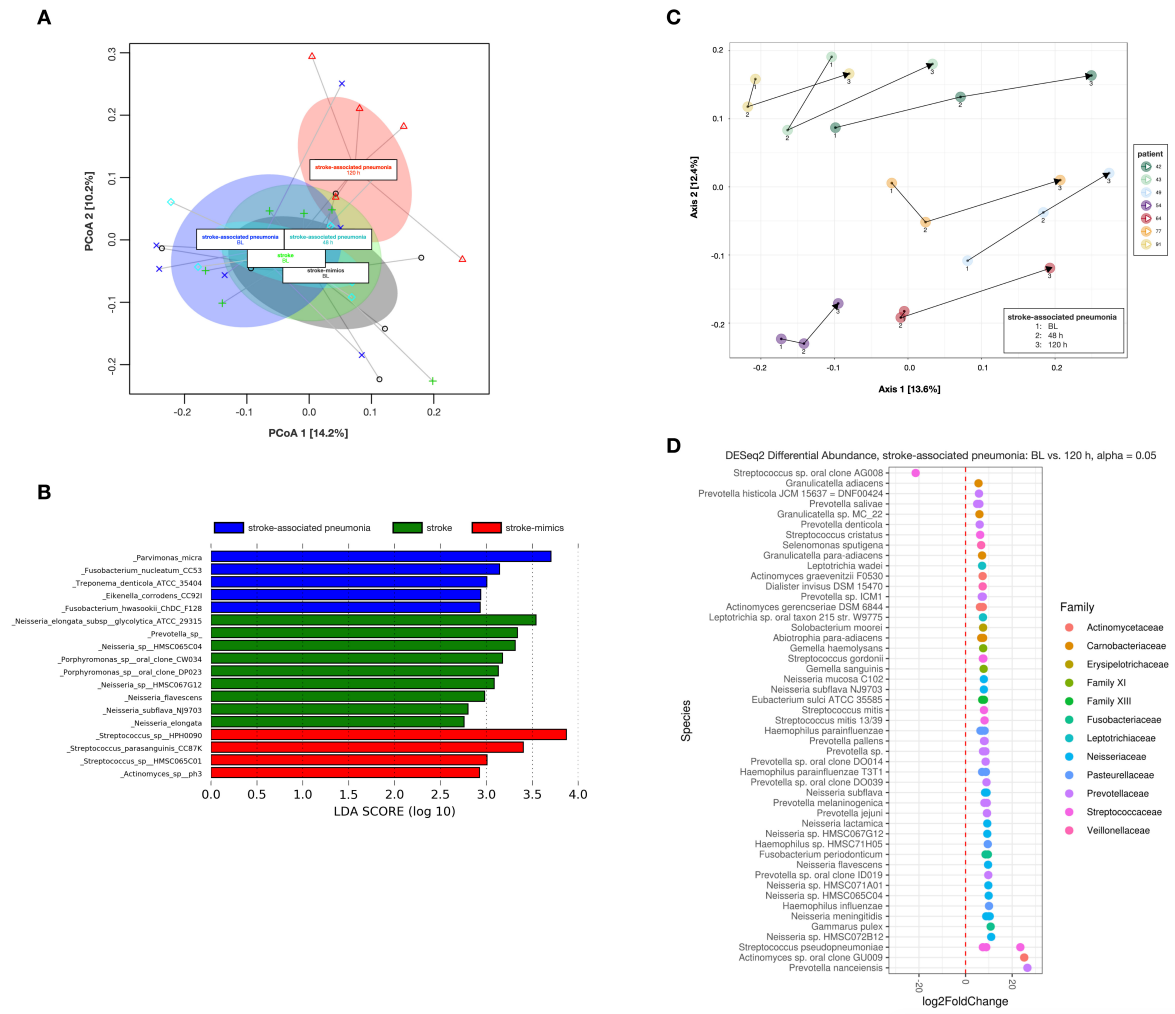
Beta diversity analysis from 16S rRNA amplicon sequencing. **(A)** Principal coordinate analysis (PCoA) of weighted UniFrac distances for analyzed samples from stroke mimics and stroke patients at baseline (BL) as well as for stroke-associated pneumonia patients at BL and at 48 and 120 h after BL. Ellipses indicate the 95% confidence interval of group centroids. **(B)** Discriminatory operational taxonomic units (OTUs) for stroke mimics, stroke patients, and stroke-associated pneumonia patients at BL, as revealed from linear discriminant analysis (LDA) effect size (LEfSe) analysis. **(C)** PCoA of unweighted UniFrac distances for the stroke-associated pneumonia patients over time at BL and at 48 and 120 h after BL (indicated by arrows for each individual patient). **(D)** Significantly differentially abundant OTUs in stroke-associated pneumonia patients between BL and 120 h after BL, as detected by DESeq2.

A total of 3,749 (mean 485 ± 187 per sample) OTUs were detected by Ion Torrent-based sequencing of V1 to V3 variable regions of bacterial 16S rRNA genes. Species richness was not significantly different between groups at BL. Also, microbial compositions based on weighted UniFrac distances (Figure 3A) showed no significant differences between stroke mimics and pneumonia patients at BL (Adonis *R*^2^ = 1.0, *p*_adj_ = 1.0) and between stroke mimics and stroke patients without pneumonia (Adonis *R*^2^ = 0.7, *p*_adj_ = 1.0). However, several OTUs were found to be discriminatory between the three groups at BL as revealed by LefSE (Figure 3B). Accordingly, the pneumonia group was characterized by the enrichment of several species within the genera *Neisseria* and *Porphyromonas* as well as *Prevotella*, accompanied by a loss of *Streptococcus* spp. as compared to the stroke mimic group.

In the analysis of longitudinal changes, the pneumonia group clustered into a distinct group after 120 h when compared to the pneumonia group (Adonis *R*^2^ = 1.6, *p*_adj_= 0.17), the stroke without pneumonia group (Adonis *R*^2^ = 1.7, *p*_adj_ = 0.04), or the stroke mimic group (Adonis *R*^2^ = 1.51, *p*_adj_ = 0.11) at BL. Significant changes over time were detected in the stroke-associated pneumonia group between BL and 120 h by DESeq2 (Figure 3C). Only one OTU assigned to *Streptococcus* sp. oral clone AG008 was significantly increased, whereas various OTUs predominantly assigned to *Actinomyces, Fusobacterium, Haemophilus, Leptotrichia, Neisseria, Prevotella*, and *Streptococcus* spp. were significantly decreased after 120 h (Figure 3D).

## Discussion

### Study Design, Study Population, and Limitations

The present study is a prospective observational study conducted on patients admitted to a stroke unit with symptoms of acute stroke within a study period of 5 months and investigated potential links between incidence of stroke-associated pneumonia and dental/oral health with a special focus on oral microbiota. From all 99 included patients, 57 patients were diagnosed with ischemic strokes, and out of these, eight patients developed pneumonia, resulting in an incidence of 14%. This is in line with the literature, where incidence rates of about 10% have been reported (6). On the other hand, there were 42 patients who were admitted to the stroke unit with symptoms of acute stroke but were not diagnosed with stroke but with other neurological diseases such as epileptic seizures, migraine with aura, vestibular neuritis, transient global amnesia, and facial nerve paresis. These so-called stroke mimics were included in this study as an “in-house” control group rather than community-dwelling elderly people because they received similar nursing standards as stroke patients and demonstrated similar neurological risk profiles as shown by the absence of significant differences with regard to comorbidities and medications. Accordingly, stroke mimics had significantly more often anamnestic history of stroke as compared to both other groups.

A major limitation of this study is the small number of pneumonia patients and the inequitable distribution of patients among the groups. The sample size for this study was based on the study period of 5 months, and within this period, all patients who were admitted to the stroke unit and fulfilled the inclusion criteria were included in this study. It was necessary to exclude patients who were already intubated at the time of admission in order to ensure sufficient dental examinations and standardized microbiological sampling. This may represent a potential selection bias, resulting in the exclusion of patients with more severe strokes as shown by the rather low NIHSS and mRS scores and also clearly limiting the case numbers of stroke (and pneumonia) patients to be included in this study. The exclusion of patients with severe strokes may also explain the high rate of stroke mimics in this study.

Despite these limitations, the study population investigated here is still representative of previous studies: Advanced age (6, 41) higher NIHSS, higher mRS, and endovascular embolectomy under general anesthesia with intubation (42, 43) are well-known risk factors for developing pneumonia after stroke. This is in line with data from Katzan et al. who found higher incidence of stroke-associated pneumonia in patients with greater stroke severity when investigating a cohort of more than 14,000 patients (8). Dysphagia and subsequent placement of nasogastric tubes as major known contributors for pneumonia were found significantly more often in pneumonia patients, a finding which is in line with numerous previous studies (6, 10, 11). Thus, nasogastric tubes offer only limited protection against development of pneumonia (44), although a strict nil-by-mouth regimen before insertion of the tube might decrease incidence of pneumonia (45).

### Immunological Data

Recently, it was reported that elevated serum levels of CRP could be a predictor for post-stroke disability and prognosis (46) as well as a diagnostic marker for stroke-associated pneumonia (47, 48). In the present study, median CRP serum levels were found to be significantly increased in pneumonia patients from the BL investigation timepoint, reaching a plateau at 48 h and decreasing again till the 120-h investigation. However, at the time of inpatient admission, median CRP levels were equal for all groups. There was a tendency for higher leukocyte counts in pneumonia patients at all investigation timepoints, which was found to be statistically significant at the 48-h investigation. Feng et al. found significantly higher leukocyte, neutrophil, and lymphocyte counts as well as higher neutrophil-to-lymphocyte ratios in patients with stroke-associated pneumonia than in stroke patients without pneumonia (49). Leukocyte counts have been proposed to be predictive of short-term (i.e., 30 days) post-stroke prognosis, most likely by reflecting the increased risk of mortality due to early infections after stroke (50).

### Dental and Oral Data

As oral diseases and insufficient oral care have been associated with incidence of aspiration pneumonia in several studies (12, 15–17), clinical parameters related to dental and oral health were investigated in the present study. The DMFT index was used to determine the burden of dental caries, and median DMFT scores were found to range between 23 and 25 with no significant differences between the groups, which is in line with recent data from the 5th German Oral Health Study (51). There were no significant differences between the groups with regard to numbers of decayed teeth, root remnants, and restored teeth, but there was still a slight tendency for more decayed teeth and root remnants in pneumonia patients, which, however, did not reach the level of statistical significance, most likely due to the limited number of pneumonia patients in this study.

Patients with stroke-associated pneumonia exhibited more missing teeth (median 22) as compared to stroke mimics (9) and stroke patients without pneumonia (10), which was found to be statistically significant between pneumonia patients and stroke mimics, but not between stroke patients with and those without pneumonia (probably also attributed to the small number of patients with stroke-associated pneumonia). The numbers of missing teeth found for pneumonia patients clearly outnumbered the numbers reported in the 5th German Oral Health Study in the respective age group (mean 17.8) (51). Tooth loss represents the end stage of oral diseases such as caries and periodontitis and, thus, is a simple, objective, and easily accessible marker for the accumulated inflammatory burden of oral disease (52, 53). A study prospectively investigating a cohort of more than 19,000 Japanese dentists also found that large numbers of lost teeth may indicate increased risk of mortality from pneumonia (54). Higher numbers of lost teeth may also reflect lower socioeconomic status (51), which in turn is associated with higher risk for incidence of stroke (55) and potentially also community-acquired pneumonia (56).

As periodontitis is known to be the major cause for tooth loss (57), higher numbers of missing teeth in the pneumonia patients may be indicative of a history of periodontal disease with its concomitant systemic effects (15, 17, 58). Dörfer et al. found a significantly higher mean PPD of 4.04 mm (SD 0.97) when investigating 303 patients with ischemic strokes as compared to 3.72 mm (SD 0.81) in 300 population controls (59). Consequently, statistically significant associations between periodontitis and ischemic stroke have been reported (60). In contrast, the present study found no significant differences in PPD among the groups. The rather shallow median pocket depth of 3 mm found in the patients with stroke-associated pneumonia may on the one hand be due to the small patient number in this group but may also result from the high numbers of missing teeth. Consequently, as a result of a median number of 22 extracted or missing teeth, the remaining teeth may just have been the ones with best prognosis (e.g., predominantly single rooted), thus not being periodontally compromised to a greater extent.

For assessing the level of oral hygiene, the API was employed as a dichotomic plaque index (31). Stroke patients and, particularly, stroke patients with pneumonia showed statistically significantly worse oral hygiene than did stroke mimics. Dörfer et al. also found statistically significantly worse oral hygiene in stroke patients than in population controls (59). Although not found statistically significant, there was a clear tendency to have even worse oral hygiene in the patients developing stroke-associated pneumonia (*p* = 0.106). As the dental examinations took place within 24 h of inpatient admission, these results cannot be attributed to insufficient oral care during hospitalization at the stroke unit. On the contrary, it is well-known that nursing-driven oral hygiene care may be of poor quality during hospitalization, particularly in dependent patients, with oral care procedures often being delegated to the least qualified members of the nursing team (61, 62). Consequently, Wagner *et al*. found that implementation of a systematic oral hygiene program significantly reduced incidence of stroke-associated pneumonia in acute stroke patients (63). Other studies even reported no cases of pneumonia in stroke patients after introduction of oral health promotion interventions such as chlorhexidine mouth rinses in conjunction with mechanical plaque removal (64, 65). Azarpazhooh et al. concluded in their systematic review that there is good evidence that improved oral hygiene reduces the occurrence of respiratory diseases among high-risk elderly in nursing homes and especially in intensive care units (66).

### Microbiological Data

Since aspiration of bacteria is an important risk factor for pneumonia, particularly in patients with insufficient oral hygiene (15, 17), carriage of pneumonia-associated pathogens or dysbiosis in the oral microbiota may contribute to development of stroke-associated pneumonia. We specifically looked at the occurrence of Gram-negative bacilli (GNB) like *Pseudomonas, Klebsiella*, and *Enterobacter* spp. that have been linked to a higher risk for pneumonia and higher mortality in acute stroke patients (67). Here, no GNB were detected with culture-dependent analysis, which may be due to the small number of denture wearers in our study (68). There were also no clear differences between the groups at the BL investigation with the most abundant genera being *Streptococcus, Neisseria, Prevotella, Rothia, Haemophilus*, and *Fusobacterium*, which were recently described to form the healthy oral core microbiota (69). For further investigation of potential differences between the groups with regard to microbial composition at BL, 16S rRNA amplicon sequencing was performed for all eight pneumonia patients with eight age- and gender-matched patients from the two other groups each. Although there were no clear compositional differences between the analyzed groups, a certain number of bacterial species within the genera *Neisseria* and *Porphyromonas* as well as *Prevotella* could be associated with pneumonia patients. As these genera are mainly linked to periodontal disease (70, 71), this finding may reflect the dental and oral data discussed above, with higher numbers of missing teeth and worse oral hygiene found in the pneumonia patients.

Interestingly, culture-dependent analysis gave a hint on some ecological changes in the pneumonia group over time with an increase in relative abundancies of *Staphylococcus, Klebsiella*, and *Candida* spp., which may be attributed to the antibiotic treatment of the pneumonia patients (9). These longitudinal changes could also be affirmed by 16S rRNA amplicon sequencing. Species and genera driving compositional changes over time were in good accordance with culture-dependent analyses. Interestingly, the only taxon that was significantly increased in patients with pneumonia between BL and 120 h was an OTU assigned to *Streptococcus* sp. oral clone AG008, which has been found in bronchoalveolar lavage from patients with ventilator-associated pneumonia (40).

## Summary

This prospective observational study investigated potential associations between dental/oral health, oral microbiota, and incidence of stroke-associated pneumonia. The major limitation of this study is the small number of patients in the pneumonia group and the inequitable distribution of patients among the groups, which clearly reduces the statistical power of some important findings. Furthermore, exclusion of intubated patients may represent a potential selection bias, resulting in exclusion of patients with more severe strokes.

Nevertheless, it was found that stroke-associated pneumonia was significantly associated with higher age, dysphagia, greater stroke severity, embolectomy treatment, insertion of nasogastric tubes, and higher CRP levels at BL. There were also trends toward higher incidence of stroke-associated pneumonia in patients with more missing teeth and worse oral hygiene level. Microbiological analyses yielded no clear differences in BL microbial composition between the patient groups. However, in the pneumonia group, there was a clear microbial shift over time, which may be attributed to the antibiotic treatment associated with pneumonia therapy.

Future studies should aim at investigating potential associations between diseased oral health and neglected oral hygiene and incidence of stroke-associated pneumonia in larger patient cohorts. In the meantime, oral hygiene programs instituted in stroke units may help reducing the incidence of pneumonia in acute stroke patients.

## Data Availability Statement

The raw data supporting the conclusions of this article will be made available by the authors, without undue reservation, to any qualified researcher.

## Ethics Statement

The studies involving human participants were reviewed and approved by internal review board of the University of Regensburg (ref. 17-806-101). The patients/participants provided their written informed consent to participate in this study.

## Author Contributions

FS, WB, AG, FC, K-AH, and JJ contributed to the study conception and design. Clinical examinations were performed by AMW, VP, AW, and DB. Data were analyzed by K-AH, AH, FC, AMW, and VP. Interpretation of the data was performed by FC, K-AH, AH, RL, JJ, WB, FS, and AG. The first draft of the manuscript was written by FC, and all authors commented on previous versions of the manuscript. All authors read and approved the final version of the manuscript.

## Conflict of Interest

The authors declare that the research was conducted in the absence of any commercial or financial relationships that could be construed as a potential conflict of interest.
